# Improving the Test-Retest Reliability of Resting State fMRI by Removing the Impact of Sleep

**DOI:** 10.3389/fnins.2017.00249

**Published:** 2017-05-08

**Authors:** Jiahui Wang, Junwei Han, Vinh T. Nguyen, Lei Guo, Christine C. Guo

**Affiliations:** ^1^School of Automation, Northwestern Polytechnical UniversityXi'an, China; ^2^QIMR Berghofer Medical Research InstituteBrisbane, QLD, Australia

**Keywords:** test-retest reliability, resting state, naturalistic paradigm, heart rate variability, sleep

## Abstract

Resting state functional magnetic resonance imaging (rs-fMRI) provides a powerful tool to examine large-scale neural networks in the human brain and their disturbances in neuropsychiatric disorders. Thanks to its low demand and high tolerance, resting state paradigms can be easily acquired from clinical population. However, due to the unconstrained nature, resting state paradigm is associated with excessive head movement and proneness to sleep. Consequently, the test-retest reliability of rs-fMRI measures is moderate at best, falling short of widespread use in the clinic. Here, we characterized the effect of sleep on the test-retest reliability of rs-fMRI. Using measures of heart rate variability (HRV) derived from simultaneous electrocardiogram (ECG) recording, we identified portions of fMRI data when subjects were more alert or sleepy, and examined their effects on the test-retest reliability of functional connectivity measures. When volumes of sleep were excluded, the reliability of rs-fMRI is significantly improved, and the improvement appears to be general across brain networks. The amount of improvement is robust with the removal of as much as 60% volumes of sleepiness. Therefore, test-retest reliability of rs-fMRI is affected by sleep and could be improved by excluding volumes of sleepiness as indexed by HRV. Our results suggest a novel and practical method to improve test-retest reliability of rs-fMRI measures.

## Introduction

Resting state functional magnetic resonance imaging (rs-fMRI) paradigm is a widely used tool to explore functional connectivity network in both healthy and clinical population (Biswal et al., 1995; Greicius et al., 2003; Fox et al., 2005; Greicius, 2008; Jafri et al., 2008; Fox and Greicius, 2010; van den Heuvel and Pol, 2010; Friston, 2011; Buckner et al., 2013; Tailby et al., 2015). The task-free nature of rs-fMRI paradigm, with low demand and high tolerance, makes it easy to standardize across study centers and conduct with subjects challenged by task performance (Greicius, 2008). Rs-fMRI has thus become a common tool in clinical studies on brain disorders, and holds great promise as imaging makers for diagnostic and prognostic uses. In addition to connectivity measures between individual brain regions, graph theory has been applied to rs-fMRI connectivity networks to measure higher order characteristics of brain networks, such as degree centrality, clustering coefficient, and modularity (van den Heuvel et al., 2008; Bullmore and Sporns, 2009; Guye et al., 2010; Hayasaka and Laurienti, 2010; He and Evans, 2010; Bullmore and Bassett, 2011; Zuo et al., 2012a).

Rs-fMRI measures, however, have not achieved the level of test-retest reliability as required by clinical imaging. The reliability of functional connectivity and graph measures derived from rs-fMRI ranges from poor to moderate (Telesford et al., 2010; Wang et al., 2011; Braun et al., 2012; Guo et al., 2012; Li et al., 2012; Patriat et al., 2013; Cao et al., 2014), where the unconstrained nature of resting state condition could have a negative impact. Without external stimulation, one problem with resting state paradigm is the excessive head motion and associated scan artifacts (Van Dijk et al., 2012; Yan et al., 2013; Vanderwal et al., 2015). It has been showed that excessive head motion reduces the reliability of fMRI measures and excluding high motion subject or volumes, or regressing out motion related artifacts could improve the reliability of rs-fMRI measures (Schwarz and McGonigle, 2011; Guo et al., 2012; Zuo et al., 2012b; Gorgolewski et al., 2013; Yan et al., 2013; Du et al., 2015).

Sleep was found to affect rs-fMRI measures in previous studies. It was reported that most subjects become drowsy and even fall asleep during resting state paradigms (Tagliazucchi and Laufs, 2014). These sleep episodes during resting state scanning are thought to be mostly non-rapid eye movement (non-REM) sleep, as more than 60 min are required to get into REM sleep (McCarley, 2007). The presence of sleep was found to affect functional connectivity and graph theoretical measures. For example, thalamocortical connectivity was found to reduce at the onset of non-REM sleep, and corticocortical connectivity increase during light sleep before getting disrupted during deep sleep (Massimini et al., 2005; Horovitz et al., 2009; Larson-Prior et al., 2009; Spoormaker et al., 2010; Koike et al., 2011; Tagliazucchi et al., 2012; Picchioni et al., 2014; Tagliazucchi and Laufs, 2014; Hale et al., 2016). Therefore, it seems possible that sleep could also affect the test-retest reliability of rs-fMRI measures, and excluding volumes of high sleepiness might improve the reliability of connectivity measures.

We here investigated this hypothesis using a test-retest fMRI dataset, where 17 participants underwent two identical fMRI sessions 3 months apart. To detect sleep during the scan, we used an established method based on simultaneous ECG recordings during the fMRI acquisition. It is well established that cardiac autonomic regulation alters between wake and different sleep stages (Burgess et al., 1997; Trinder et al., 2012; Tobaldini et al., 2013). Compared with wake condition, non-REM sleep often incurs a marked decrease in heart rate and increase in HRV. The changes start from sleep onset, or when subjects feel drowsy, and continue throughout the non-REM sleep stage. This suggests a general cardiovascular output reduction and a transfer from predominant sympathetic to parasympathetic cardiac modulation during non-REM sleep (Toscani et al., 1996; Elsenbruch et al., 1999; Trinder et al., 2001; Busek et al., 2005; Carrington et al., 2005; de Zambotti et al., 2011, 2014; Cabiddu et al., 2012; Boudreau et al., 2013; Chouchou and Desseilles, 2014; Cellini et al., 2016). HRV could thus be used to detect sleep or drowsiness.

In sleep studies, electroencephalogram (EEG) is recognized as gold standard to identify sleep stages (Rechtschaffen and Kales, 1968; Iber et al., 2007). Nevertheless, it is hard for subjects to fall asleep with EEG scalp on. Lv et al. identified sleep state using HRV derived from peripheral pulse signals, and observed consistent brain network properties compared to those derived from EEG based studies (Lv et al., 2015). Moreover, HRV measures are widely used, solely or combined with other physiological signal measures, as features in machine learning models to predict and detect the fatigue and sleepiness of drivers. The classification accuracy could reach over 90% (Lal and Craig, 2001; Borghini et al., 2012; Sahayadhas et al., 2012; Abbood et al., 2014). Furthermore, compared to other biosignals used for sleep detection, such as EEG and pupillometry (Abbood et al., 2014), simultaneous recording of cardiac signals, using either ECG or pulse oximetry, is more easily and routinely implemented in fMRI experiments.

Here, we used HRV derived from the ECG to index the level of alertness and sleepiness continuously for each fMRI volume. We then examined the effect on the test-retest reliability of connectivity measures when the volumes of the most extreme HRV values were excluded. To derive a more general conclusion, we used two different HRV measurements—the root mean square of successive difference of normal-to-normal intervals (RMSSD) (Neumann et al., 1941; Malik, 1996) and cardiac vagal index (CVI) (Toichi et al., 1997) to index the level of sleep, independently, and assessed test-retest reliability at both individual unit- and scan-wise levels (Guo et al., 2012).

## Materials and methods

### Participants

Twenty right-handed participants (11 females, 9 males; aged between 21 and 31 years; mean age 27 ± 2.7 years) participated in the study. The participants were recruited from the University of Queensland and provided written informed consent. Participants received a small monetary compensation ($50) for their participation in the study. The study was approved by the human ethics research committee of the University of Queensland and was conducted according to National Health and Medical Research Council guidelines.

### Experimental paradigm

The experiment comprised two scanning sessions. For each session, participants underwent an 8-min resting state fMRI exam with eyes closed, and then freely viewed a 20-min short movie “*The Butterfly Circus*.” Resting state condition was always acquired first to avoid potential effect of movie viewing experience on resting state brain activity, and also to reduce the likelihood of fatigue and sleep during resting state. *The Butterfly Circus* is a short film that depicts an intense, emotionally evocative story of a man born without limbs who is encouraged by the showman of a renowned circus to reach his own potential. The movie is live action, color, and shot in high definition. Additional details of the experiment were previously reported (Nguyen et al., 2016b; Wang et al., 2017).

Three months after the first scan session (Session A), participants returned for the second imaging session (Session B) involving an identical protocol of resting state and movie viewing paradigms. Three participants were excluded from the reliability analysis: one was due to technical problems during data recordings and the other two did not return for the second session. Hence, test-retest reliability analyses were performed on data from the 17 participants who finished both scan sessions.

### Functional image acquisition and preprocessing

Functional and structural images were acquired from a whole-body 3-Tesla Siemens Trio MRI scanner equipped with a 12-channel head coil (Siemens Medical System, Germany). Functional images were acquired using a single-shot gradient-echo Echo Planar-Imaging (EPI) sequence with the following parameters: repetition time (TR) 2,200 ms, echo time (TE) 30 ms, flip angle (FA) 79°, Field of View (FOV) 192 × 192 mm, pixel bandwidth 2,003 Hz, a 64 × 64 acquisition matrix, 44 axial slices, and 3 × 3 × 3 mm^3^ voxel resolution. A high-resolution T1-weighted MPRAGE structural image covering the entire brain was also collected for each participant with the following parameters: TE = 2.89 ms, TR = 4,000 ms, FA = 9°, FOV = 240 × 256 mm, and voxel size 1 × 1 × 1 mm^3^.

Functional images were preprocessed using Statistical Parametric Mapping toolbox (SPM12, Welcome Department of Imaging Neuroscience, Institute of Neurology, London) and a toolbox for Data Processing & Analysis for Brain Imaging (DPABI) (Yan et al., 2016) implemented in Matlab (Mathworks, USA). The first five volumes of each EPI sequence were discarded to allow scanner equilibrium to be achieved. The remaining functional images were slice-time corrected, realigned, co-registered to the T1 structural image of each individual subject, and normalized to the Montreal Neurological Institute (MNI) space without additional smoothing. The images were further regressed out of nuisance signals, bandpass filtered (0.0083–0.15 Hz) and detrended. Nuisance signals include principle components of WM and CSF signals derived using the CompCor method (Behzadi et al., 2007) and Friston-24 motion parameters (Friston et al., 1996; Yan et al., 2013). Additional preprocessing details were previously reported (Wang et al., 2017). After preprocessing, there are total 215 and 530 volumes for resting state and natural viewing conditions, respectively.

### Heart rate variability

ECG signals were recorded using Brain Products system (http://www.brainproducts.com/). The leads were placed on the back, and the signals were recorded at the sampling rate of 5,000 Hz. Heart beats were first detected automatically using the detection algorithm implemented in QRSTool software (Allen et al., 2007). The detected heart beats were then visually checked and the misidentified ones were manually corrected. Inter-beat intervals (IBI) were then calculated as the time intervals between two successive individual beats. Using HRVAS toolbox (Ramshur, 2010), the resultant IBIs were further cleaned and processed (ectopic values removed, interpolated, and detrended). Finally, the IBIs were used to derive HRV measures: the root mean square of successive difference of IBIs (RMSSD) and Tochi cardiac vagal index (CVI). These two measures are believed to primarily reflect parasympathetic function (Neumann et al., 1941; Malik, 1996; Toichi et al., 1997).

Next we used sliding windows to derive continuous HRV (Guo et al., 2016). Sliding windows were centered in the middle of each TR, moving forward in steps of 1 TR. HRV measures were calculated using the IBIs within each window. We examined a series of window lengths: 4, 8, 12, …, 50 s, and the proper window length was chosen based on the following criteria: (1) the time-varying HRV is highly consistent with the overall HRV, measured as the ratio of time-varying HRV averaged across all windows and subjects to the overall HRV averaged across all subjects (Thong et al., 2003); (2) the test-retest reliability of the time-varying HRV measures is good. We finally chose RMSSD and CVI with the window length of 16 s for the following analyses, because they are highly consistent with the whole scan HRV (>0.95), relatively reliable (RMSSD: scan-wise ICC: 0.8, unit-wise ICC: 0.672; CVI: scan-wise ICC: 0.693, unit-wise ICC: 0.53. Method of calculating unit- and scan-wise ICC is described below in *Test-retest reliability*), and could still provide satisfactory time resolution.

This continuous HRV was then used as an estimate of the level of sleepiness during each TR. We used a relative threshold of 50% to select the top 50 percentile sleepiest (highest HRV values, sleepy-0.5) or most alert (lowest HRV values, alert-0.5) volumes from each session in the reliability analyses. To exclude any non-specific effect due to volume selection, we created a control condition by randomly selecting 50% volumes and taking the average from 5,000 randomizations (random-0.5). To ensure the robustness of our results to the selection threshold of certain state and the window lengths of time varying HRV, we performed additional reliability analyses: (1) using a serial of additional thresholds of data inclusion (0.9, 0.8, 0.7, 0.6, 0.4, 0.3) when HRV was derived using 16 s sliding window; (2) using a serial of window lengths (4, 8, 12, …, 50 s) to derive the time-varying HRV, then performed reliability analyses for sleepy-0.5 and alert-0.5 conditions. We then used RMSSD derived from 16 s sliding window to derive continuous HRV for movie viewing data, and examined the effects of sleepiness on test-retest reliability in natural viewing conditions. To make the analyses on resting state and natural viewing conditions more comparable, we performed additional analyses on an 8-min segment of the natural viewing data, which matched the duration of the resting state sessions.

### ROI-based functional connectivity analysis

We first performed functional connectivity analysis using a previous established atlas: the 200 ROI atlas based on Craddock 2012 parcellation (Craddock et al., 2012), as it provides good cortical and subcortical coverage with fine divisions.

ROIs' time series were extracted as the mean signal across all voxels within each ROI from preprocessed fMRI data. Pearson correlation was then computed between each pair of ROIs' time series using the sleepy-0.5, alert-0.5, random-0.5 and whole data separately, resulting in four 200 × 200 connectivity matrices for each subject for each session. For each matrix, the correlation coefficients were transformed to z-scores using Fisher's transformation, averaged across all subjects for each condition, and then reverted to Pearson's r values to derive group-level connectivity matrices (Zuo et al., 2012a; Vanderwal et al., 2015). To quantitatively evaluate the differences between connectivity matrices at different alertness levels, we performed paired *t*-test across subjects on the connectivity matrices between sleepy-0.5 and alert-0.5 conditions. The results were thresholded using FDR-corrected *p* < 0.05.

### Graph theoretical analysis

We further derived graph theoretical measures from the ROI connectivity matrices. We produced weighted adjacent matrices by thresholding the fully connected ROI matrices: suprathreshold connections (edge) retained their correlation coefficients denoting edge weights, whereas subthreshold edges were assigned values of 0. To ensure robustness of the threshold chosen, we repeated our analyses using a serial of thresholds (T_r_ = 0.1, 0.3, and 0.5).

We focused on two graph metrics that have been shown to be reliable: degree centrality and clustering coefficient (Braun et al., 2012; Guo et al., 2012; Andellini et al., 2015; Du et al., 2015; Wang et al., 2017). These graph metrics were derived from the weighted adjacency matrices using Brain Connectivity Toolbox (Rubinov et al., 2009). Degree centrality measures the connectedness of each node, computed as the weighted sum of all the edges connected to the node. Clustering coefficient measures the likelihood of the nodes tending to cluster together, calculated as the fraction that the number of edges actually exist to the number of all edges possibly exist. To examine the differences between graph measures with different sleepiness levels, we performed paired *t*-test across subjects on the graph measures between sleepy-0.5 and alert-0.5. The results were thresholded using an FDR-corrected *p* < 0.05.

### Test-retest reliability

In this paper, we assessed test-retest reliability using intraclass correlation coefficient (ICC) (Shrout and Fleiss, 1979; McGraw and Wong, 1996; Caceres et al., 2009). A one-way ANOVA was applied to the measures of the two scan sessions across subjects, to calculate between-subject mean square (*MS*_*b*_) and within-subject mean square (*MS*_*w*_). ICC values were then calculated as:

$$\begin{array}{l} ICC=\frac{MS_{b}-MS_{w}}{MS_{b}+\left(d-1\right)MS_{w}} \end{array}$$

where *d* = the number of observations per subject. For every functional connectivity measure, we assessed reliability at both individual unit-wise and scan-wise levels. Unit-wise reliability is commonly reported in the literature (Shehzad et al., 2009; Wang et al., 2011; Braun et al., 2012; Guo et al., 2012; Zuo et al., 2012a; Birn et al., 2013; Liao et al., 2013). Here, one ICC value was calculated for each measurement unit, such as the HRV value of each window, the connectivity score of each ROI pair (edge), or graph metric of each ROI (node). Unit-wise ICC was then produced by averaging the ICC values for all measurement units across the windows or the network to represent reliability at individual level. Additionally, we reported scan-wise reliability, which estimates the reliability of the mean measurement derived from the entire scan session or the whole graph (Guo et al., 2012). Here, a single ICC value was calculated for the mean HRV values, mean connectivity scores or graph metric averaged across all windows of the whole scan, or edges or nodes of the network.

The reliability results are referred as excellent (ICC > 0.8), good (0.79 > ICC > 0.6), moderate (0.59 > ICC > 0.4), fair (0.39 > ICC > 0.2), and poor (ICC < 0.2) (Guo et al., 2012).

### Statistical test

We tested whether ICC values of sleepy and alert conditions were significantly different from corresponding random condition, at both unit- and scan-wise levels. We performed one-tailed permutation test by comparing the true ICC value against the distribution of ICCs from the permuted random conditions (details see *Heart Rate Variability*). A 95% CI for each permutation test was calculated as the highest value (right-tailed test) or lowest (left-tailed test) with an alpha level of 0.05 (Lamotte and Volaufova, 1999; Ernst, 2004).

### Head motion

We also examined the amount of head motion during different levels of sleepiness, using framewise displacement proposed by Power et al. (2012). Framewise displacement is a scalar quantity defined as: *FD*_*i*_ = |Δ*d*_*ix*_| + |Δ*d*_*iy*_ + |Δ*d*_*iz*_| + |Δα_*i*_| + |Δβ_*i*_| + |Δγ_*i*_|, where *d*_*ix*_, *d*_*iy*_ and *d*_*iz*_ are translational displacements along X, Y and Z axes, respectively; α_*i*_, β_*i*_ and γ_*i*_ are rotational angles of pitch, yaw and roll, respectively; Δ*d*_*ix*_ = *d*_(*i*−1)*x*_ + *d*_*ix*_, Δ*d*_*iy*_ = *d*_(*i*−1)*y*_ + *d*_*iy*_, Δ*d*_*iz*_ = *d*_(*i*−1)*z*_ + Δγ_*i*_ = α_(*i*−1)_ + Δβ_*i*_, Δβ_*i*_ = Δγ_*i*−1_ + β_*i*_, Δγ_*i*_ = γ_(*i*−1)_ + γ_*i*_. Rotation displacements were converted from degrees to millimeters of distance on a sphere surface (radius: 50 mm, assumed to be the radius of a head). One spike was counted when *FD*_*i*_ was greater than 0.3 mm (Yan et al., 2013; Vanderwal et al., 2015). We calculated the frequency of spikes as the number of spikes per volume and compared it between the different alert levels using paired *t*-test across subjects. We didn't find any significant influence of sleep on head motion.

## Results

### Heart rate variability during resting state fMRI

**Figure 1:**
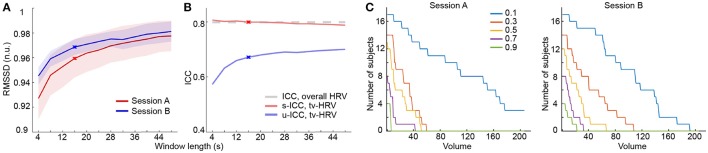
**Heart rate variability analysis based on RMSSD. (A)** Normalized RMSSD (RMSSD (n.u.), n.u. stands for “normalized units”) averaged across windows and subjects using different window length in both sessions. **(B)** ICCs of time-varying HRV (tv-HRV) using different window length at both unit- and scan-wise levels. The ICC of overall HRV is indicated by the dashed line. The window chosen to derive the main results (16 s) is signified by crosses. **(C)** The number of subjects who successively stayed alert with scanning progression using a serial of selection threshold of sleepiness (signified by different colors). The appearance of consecutive 5 sleepy volumes was used as dropout criterion.

HRV is modulated by both sympathetic and parasympathetic nervous systems (Acharya et al., 2006), while the parasympathetic modulation is predominant at rest. We here used two common HRV metrics reflecting mainly parasympathetic modulation—RMSSD (Malik, 1996) and CVI (Toichi et al., 1997) to measure the overall and time-varying HRV during rs-fMRI. The overall HRV measures showed good test-retest reliability (RMSSD: 0.799; CVI: 0.681). Then we used a sliding window method to derive time-varying HRV metrics based on RMSSD and CVI (Guo et al., 2016). With proper window length, time-varying HRV measures were highly consistent (>0.95) with overall HRV metrics [Figure 1A; SFigure 1A; results based on RMSSD are presented in main text (Figures), and those based on CVI are in Supplementary Materials (SFigure)], and showed moderate to good test-retest reliability (RMSSD: scan-wise ICC: 0.8, unit-wise ICC: 0.672; CVI: scan-wise ICC: 0.693, unit-wise ICC: 0.53; Figure 1B; SFigure 1B).

It is well established that HRV increases as one gets drowsier, which has been used to detect driver alertness (Lal and Craig, 2001; Borghini et al., 2012; Abbood et al., 2014). Here, we used the time-varying HRV measures as a way to index sleepiness during resting state fMRI scans. Consistent with previous work using EEG for sleep detection (Tagliazucchi and Laufs, 2014), the number of subjects who stayed alert decreased as the scan time increased (Figure 1C; SFigure 1C).

### Reliability of functional connectivity measures affected by sleep

**Figure 2:**
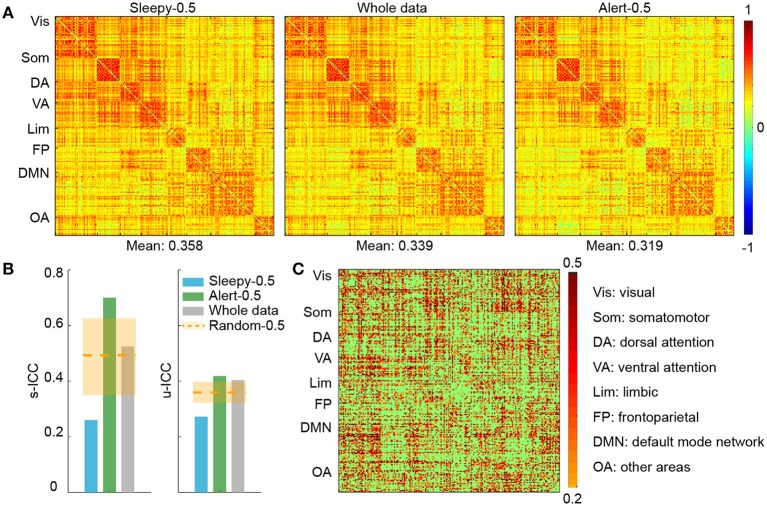
**ROI connectivity matrix analysis of alert and sleepy conditions based on RMSSD. (A)** Group-level connectivity matrices derived from the sleepy-0.5, whole-scan and alert-0.5 conditions during session A. ROIs were organized according to the 7-network system (Yeo et al.), as labeled on the left of each panel. The mean connectivity strength of each condition is indicated on the bottom of each matrix. The connectivity matrices in session B are very similar to those in session A, and thus not presented. **(B)** Functional connectivity ICCs during resting state at both scan- (left panel) and unit-wise (right panel) levels. Unit-wise ICC was averaged across ROI pairs. Orange dashed lines indicate the average ICC values of the random-0.5 conditions, and the shaded boxes indicate their distributions—upper and lower bounds marking the 95 and 5 percentiles, respectively. Values outside the boxes are significantly different from the random conditions (one-tailed permutation test, *p* < 0.05). **(C)** Unit-wise ICC differences between alert-0.5 and sleepy-0.5 conditions (warm color: alert-0.5 > sleepy-0.5; cool color: alert-0.5 < sleepy-0.5). Differences greater than 0.2 are displayed.

To examine the effect of sleep on functional connectivity measures and their test-retest reliability, we performed connectivity and reliability analyses using either the 50% of data when subjects were most alert (alert-0.5) or the 50% when subjects were sleepiest (sleepy-0.5). We chose a parcellation scheme of 200 ROIs (Craddock et al., 2012), which covers the entire cortical and subcortical regions, and organized the ROIs into seven networks (Yeo et al., 2011). The seven networks are: visual, somatomotor, dorsal attention, ventral attention, limbic, frontoparietal, default, and other areas (including parts of cerebellums, thalamus, brainstems, and caudate). Overall, group averaged functional connectivity matrices derived from alert-0.5, sleepy-0.5, and whole data conditions showed similar patterns (Figure 2A; SFigure 2A). Direct comparison between sleepy-0.5 and alert-0.5 conditions did not detect significant differences (paired *t*-test, FDR-corrected *p* < 0.05).

We then assessed whether the sleepiness affected the reliability of functional connectivity measures. Following previous studies (Guo et al., 2012), unit- and scan-wise ICC measures were used to quantify the test-retest reliability of functional connectivity measures during sleepy-0.5 and alert-0.5 conditions, respectively. Unit-wise ICC refers to that ICC was calculated for each pair of ROI connection, and scan-wise ICC derived from connectivity strengths averaged across the whole connectivity matrix. As reliability decreases with less data volumes (Birn et al., 2013), we created a control condition of 50% randomly selected volumes (random-0.5) to compare with the alert-0.5 and sleepy-0.5 conditions. Compared to the random-0.5 condition, the sleepy-0.5 condition resulted in significantly lower ICC and the altert-0.5 condition produced significantly higher ICC for both unit- and scan-wise measures (permutation test, *p* < 0.05; Figures 2B,C; SFigure 2B; Table 1; Stable 1), suggesting that sleepiness during resting state scans reduced the reliability of functional connectivity measures. Even directly compared to the whole data condition, the alert-0.5 condition yielded higher reliability. The ICC values increased by 3.7 and 33.4% at individual unit- and scan-wise levels, respectively (Figure 2B; SFigure 2B), further confirming that the volumes with high sleepiness were associated with low reliability.

**Table 1:** **One-tailed permutation tests of the differences in resting state reliability between the sleepy-0.5 or alert-0.5 and the random-0.5 conditions, based on RMSSD**.

		**Unit-wise**			**Scan-wise**		
		**Random**	**Sleepy**	**Alert**	**Random**	**Sleepy**	**Alert**
Functional connectivity	ICC	[0.321, 0.397]	0.273	0.418	[0.349, 0.624]	0.26	0.7
	*p*	–	0.0004	0.0038	–	0.0048	0.003
Clustering coefficient	ICC	[0.314, 0.550]	0.216	0.636	[0.341, 0.628]	0.228	0.707
	*p*	–	0.0024	0.001	–	0.0034	0.0042
Degree centrality	ICC	[0.338, 0.519]	0.257	0.584	[0.351, 0.625]	0.252	0.699
	p	–	0.002	0.0014	–	0.0044	0.0032

*Graph theoretical metrics were derived with T_r_ = 0.1. ICC and p values are listed for each condition. ICCs of random condition are indicated using upper and lower bounds marking the 95 and 5 percentiles of the random distribution, respectively*.

### Reliability of graph theoretical measures affected by sleep

We then assessed the effect of sleep on graph theoretical measures. We focused on the graph metrics known to be reliable: clustering coefficient and degree centrality (Braun et al., 2012; Guo et al., 2012; Wang et al., 2017). To ensure the robustness of our results, graph theoretical measures were derived using a broad range of thresholds: T_r_ = 0.1, 0.3, 0.5. Overall, the level of sleepiness did not affect graph theoretical measures (SFigure 3). There was a slight decrease with alert-0.5 condition, but this decrease was not statistically significant (SFigure 3; paired *t*-test, FDR-corrected *p* < 0.05).

**Figure 3:**
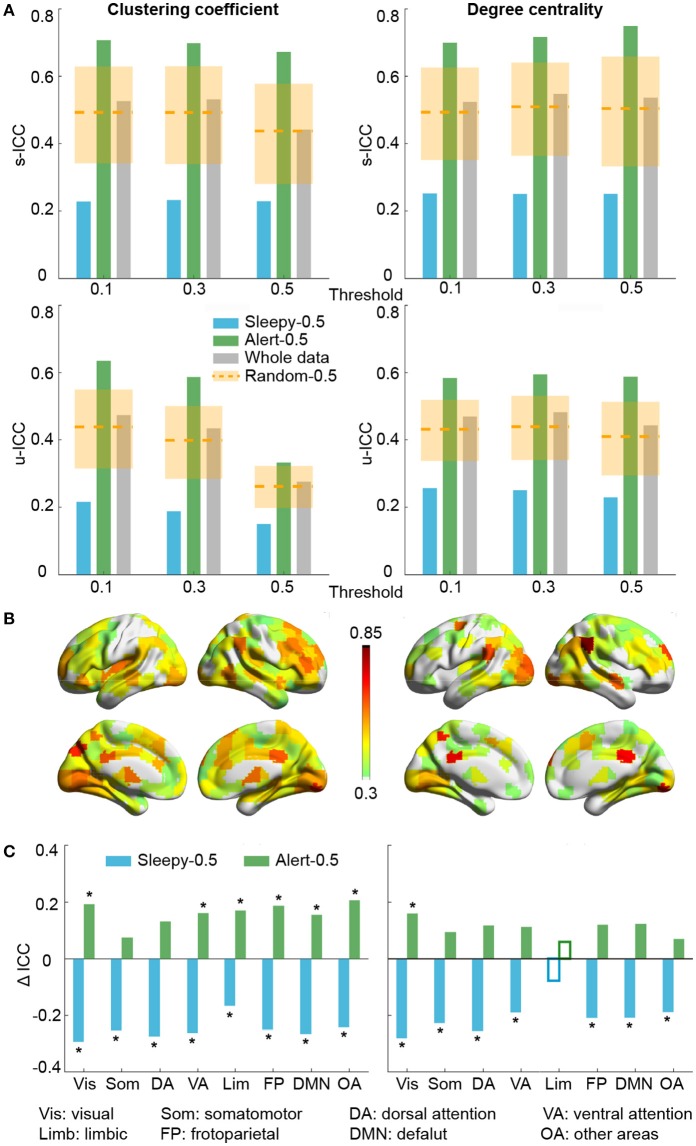
**Test-retest reliability analysis using graph theoretical measures, based on RMSSD. (A)** Average unit-wise (upper panel) and scan-wise (lower panel) ICCs during resting state across three thresholds (T_r_ = 0.1, 0.3, 0.5). Orange dashed lines indicate the average ICC values of the random-0.5 conditions, and the shaded boxes indicate their distributions—upper and lower bounds marking the 95 and 5 percentiles, respectively. Values outside the boxes are significantly different from the random conditions (one-tailed permutation test, p < 0.05). **(B)** Unit-wise ICC differences between sleepy-0.5 and alert-0.5 conditions (warm color: alert-0.5 > sleepy-0.5; cool color: alert-0.5 < sleepy-0.5). Differences greater than 0.3 are displayed. **(C)** Unit-wise ICC difference between sleepy-0.5 or alert-0.5 and the whole data at network level, which is represented using mean across ROIs within each network. Solid bars indicate significant differences compared to the random-0.5 condition (one-tailed permutation test, FDR-corrected *p* < 0.05). Asterisks indicate ICC changes over 30% relative to the whole data condition. Results in **(B,C)** were generated using T_r_ = 0.1.

We then assessed the test-retest reliability of each graph measure. Similar to functional connectivity, ICCs derived from the sleepy-0.5 condition were significantly lower than those from the random-0.5 condition, while those from the alert-0.5 condition were significantly higher, irrespective of the threshold used (permutation test, *p* < 0.05; Figure 3A; SFigure 4A; Table 1; STable 1). Furthermore, ICC values derived from the alert-0.5 conditions were also improved when compared to those from the whole data condition, which increased by 29.8% at unit-wise and 37.7% at scan-wise levels averaged across both graph measures and all three thresholds applied (Figure 3A; SFigure 4A).

To examine whether these changes in reliability was specific to certain brain networks, we compared unit-wise ICCs across each brain network (Figure 3B; SFigure 4B). The average reliability was calculated as the arithmetic mean across ROIs included in each network. Under alert-0.5 condition, the ICCs increased by more than 30% in most networks for clustering coefficient, and over 25% for degree centrality (Figure 3C; SFigure 4C). To ensure the robustness of the improvement, we also used the median ICCs to represent the average reliability within each network, and observed consistent results (SFigure 7).

### Test-retest reliability with different data selection thresholds

**Figure 4:**
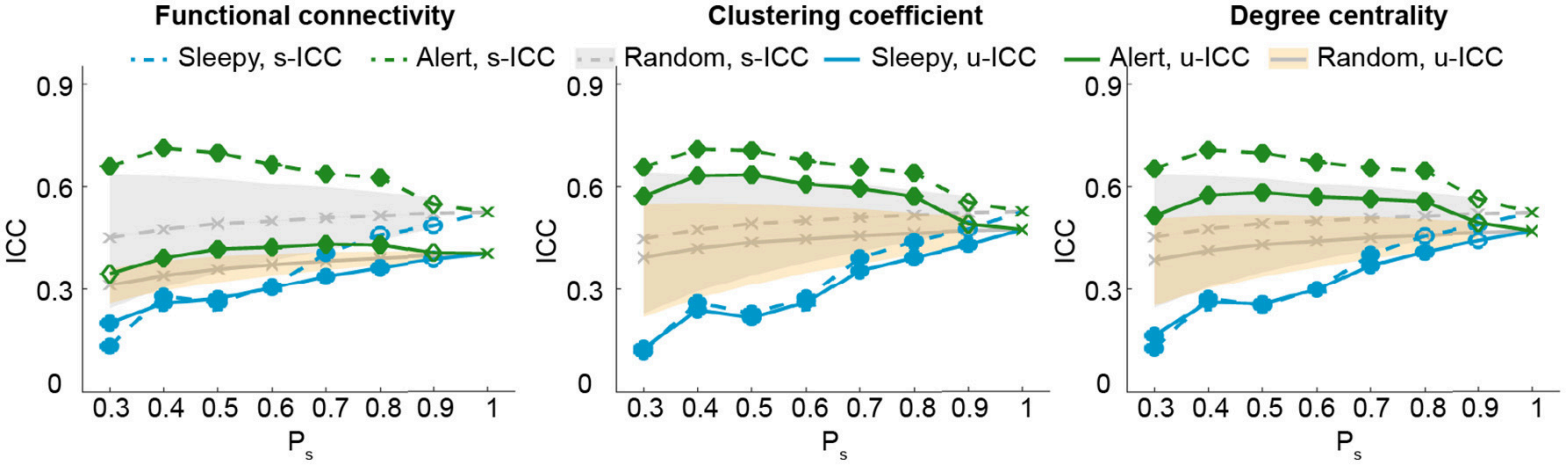
**Test-retest reliability analysis using a serial of volume selection percentiles (P_**s**_), based on RMSSD**. The shades indicate the distribution of the ICCs derived from random condition—upper and lower bounds marking the 95 and 5 percentiles, respectively. Values outside the shades are significantly different from the random conditions, and represented using solid markers (one-tailed permutation test, *p* < 0.05). Results of clustering coefficient and degree centrality were obtained from T_r_ = 0.1.

So far, our results show that the test-retest reliability is improved when excluding the top 50 percentile data of high sleepiness. We then asked what percentage of volumes selection is optimal for improving test-retest reliability. We tested a range of percentiles to select volumes (Figure 4; SFigure 5). When volumes were randomly selected (random conditions), ICC value decreased with less volumes included (Birn et al., 2013). However, when specifically selecting volumes based on HRV, ICCs increased significantly and continuously with less volumes of high sleepiness included in the calculation, till as much as 60% sleepy volumes were excluded (Figure 4; SFigure 5), suggesting that the detrimental effect of sleepiness on reliability outweighed the effect of reduced volumes. In practice, however, it might be desirable to remove the minimal amount of data volume and we found 20% was the least amount of sleepy volumes required to significantly improve test-retest reliability for all three measures (Figure 4; SFigure 5).

### Reliability of natural viewing paradigm not affected by sleep

As we showed previously that the reliability of connectivity measures were higher during natural viewing than resting state condition (Wang et al., 2017), we then asked whether it could be further improved by this approach.

**Figure 5:**
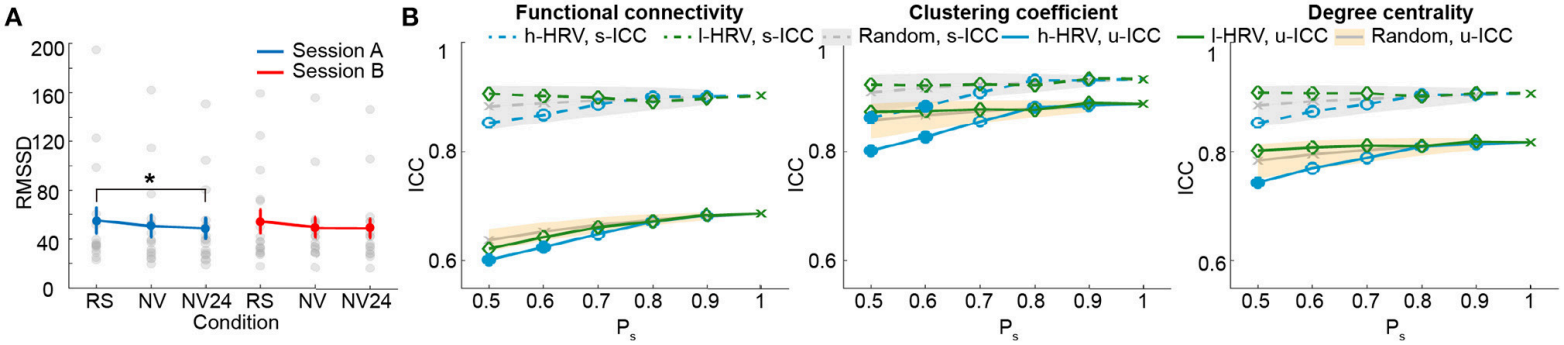
**Analysis for natural viewing conditions based on RMSSD. (A)** RMSSD values derived from resting state (RS), natural viewing (NV), and the 24th segment of natural viewing data (NV24). Data from each subject is signified by the gray dots. Error bars indicate the standard error of the mean. **(B)** Test-retest reliability analysis using a serial of P_s_. The shades indicate the distribution of the ICCs derived from random condition—upper and lower bounds marking the 95 and 5 percentiles, respectively. Values outside the shades are significantly different from the random conditions, and represented using solid markers (one-tailed permutation test, *p* < 0.05). Results of clustering coefficient and degree centrality were obtained from T_r_ = 0.1.

We first examined the measures of HRV during natural viewing. On average, HRV during natural viewing reduced slightly, but this reduction was not significant (paired *t*-test, *p* < 0.05; Figure 5A). We further derived HRV from the most engaging movie segment based on our previous study (Wang et al., 2017), and found that HRV during this segment was significantly lower than resting state in session A (paired *t*-test, *p* < 0.05; Figure 5A). Furthermore, HRV measures were more reliable during natural viewing (0.928) than resting state (0.799), similar to our findings with functional connectivity measures (Wang et al., 2017).

We then compared the unit- and scan-wise ICCs of functional connectivity measures. The results derived from the 8-min segment were similar to the results using the entire natural viewing data (Figure 5B; SFigure 8; Table 2). While the reliability of conditions with higher HRV level decreased, these changes were much smaller than the ones during resting state conditions. And we did not find consistent and significant increases in reliability with the low HRV conditions (Figure 5B; SFigure 8).

**Table 2:** **One-tailed permutation tests of the difference in movie viewing reliability between the sleepy-0.5 or alert-0.5 and the random-0.5 conditions, based on RMSSD**.

		**Unit-wise**			**Scan-wise**		
		**Random**	**Sleepy**	**Alert**	**Random**	**Sleepy**	**Alert**
Functional connectivity	ICC	[0.617, 0.658]	0.602	*0.622*	[0.841, 0.922]	*0.854*	*0.907*
	*p*	–	0.0024	*0.1038*	–	*0.1178*	*0.1774*
Clustering coefficient	ICC	[0.824, 0.889]	0.801	*0.874*	[0.873, 0.941]	0.863	*0.924*
	*p*	–	0.006	*0.2194*	–	0.022	*0.2575*
Degree centrality	ICC	[0.750, 0.814]	0.743	*0.802*	[0.846, 0.922]	*0.853*	*0.909*
	*p*	–	0.0264	*0.176*	–	*0.0894*	*0.1572*

*Graph theoretical metrics were derived with T_r_ = 0.1. ICC and p values are listed for each condition. ICCs of random condition are indicated using upper and lower bounds marking the 95 and 5 percentiles of the random distribution, respectively. Non-significant results are in italic*.

## Discussion

In this study, we examined the effect of sleep on test-retest reliability of rs-fMRI connectivity measures. By excluding volumes acquired when participants were sleepy, we could improve the reliability of network connectivity measures during rs-fMRI paradigm. The improvement of test-retest reliability is robust with removal of as little as 20% of volumes. Noticeably, this improvement on ICC outweighs the opposing effect from reduced volume (Birn et al., 2013). Overall, our results provide a novel and practical way to improve test-retest reliability of rs-fMRI paradigm.

The test-retest reliability of rs-fMRI measures ranges between poor to moderate (Telesford et al., 2010; Wang et al., 2011; Braun et al., 2012; Guo et al., 2012; Li et al., 2012; Patriat et al., 2013; Cao et al., 2014). Many factors contribute to the moderate reliability, including poor signal-to-noise ratio of the blood oxygenation level-dependent (BOLD) signal, excessive head motion, physiological noise, and so on. Previous work has found that test-retest reliability can be improved by removing volumes or subjects with excessive motion, and regressing out motion related artifacts (Schwarz and McGonigle, 2011; Guo et al., 2012; Zuo et al., 2012b; Gorgolewski et al., 2013; Yan et al., 2013; Du et al., 2015). Now we showed that the presence of drowsiness and sleep during scanning is another factor affecting rs-fMRI measures and their reliability. Due to acoustic noise, fatigue, and the lack of stimulation, it is common that subjects fall asleep during rs-fMRI scans (Tagliazucchi and Laufs, 2014). Sleep was found to be associated with changes in brain network, urging caution when interpreting functional connectivity measures during resting state (Massimini et al., 2005; Larson-Prior et al., 2009; Spoormaker et al., 2010; Koike et al., 2011; Picchioni et al., 2014; Tagliazucchi and Laufs, 2014; Hale et al., 2016). Some methods were proved to be effective to prevent subjects from falling asleep, such as requiring subjects to keep eyes open or fixed on a cross (Patriat et al., 2013; Zou et al., 2015), and their test-retest reliability are higher than resting state with eyes closed. However, this impact on connectivity measures and their test-retest reliability appears to differ across brain networks (Patriat et al., 2013; Zou et al., 2015).

Previous studies report decreases in heart rate and increases in HRV at the transition from wake to non-REM sleep. These changes have thus been widely used to detect sleepiness in real life situations (Lal and Craig, 2001; Borghini et al., 2012; Abbood et al., 2014). While previous studies used long ECG data to derive HRV (5 min to 24 h), recent studies have used shorter duration (10–250 s) to improve the temporal resolution (Thong et al., 2003; Salahuddin et al., 2007; Udi et al., 2011; Chang et al., 2013; Valenza et al., 2014; Guo et al., 2016; Massaro and Pecchia, 2016; Nguyen et al., 2016a). In this study, we examined the robustness and reliability of HRV metrics derived using different window lengths. For both RMSSD and CVI measures, the metrics derived using short data durations are highly consistent (>0.95) with the ones derived using the whole 8-min data, and RMSSD achieves good test-retest reliability with the window length of as short as 6 s. These analyses support the use of short-term HRV as time-varying measures. It is increasingly recognized that physiological fluctuations could introduce noise in fMRI signals. It is possible that higher HRV might contribute to greater fMRI noise. Removal of noisy volumes could thus lead to an improvement in reliability. In our current experimental design, it is not possible to discern between the contributions of physiological noise and sleepiness. Irrespective of the source, excluding volumes of high HRV could still provide a valid strategy to improve test-retest reliability of rs-fMRI connectivity.

We excluded volumes of high or low HRV for connectivity and test-retest reliability analyses. This approach is similar to the motion scrubbing method proposed to reduce the impact of motion artifacts (Power et al., 2012). In some study, an average of 58% data were scrubbed for a cohort of children where motion is problematic. In our dataset, after excluding 50% sleepiest volumes, we found ICC values increased by 24.9% (0.108) at the unit-wise level and 36.4% (0.187) at the scan-wise level averaged across the three measures we examined (functional connectivity, clustering coefficient and degree centrality), and across all three thresholds for graph measures. The test-retest reliability also improved in higher order brain networks, such as dorsolateral prefrontal cortex, angular gyrus, and cingulate cortex (Figure 3; SFigure 4), reflecting the impact of sleep on these brain regions. In our main analyses, the volume-wise sleepiness level was identified using the time-varying HRV derived from 16 s sliding window, which was chosen based on a tradeoff between time resolution and the robustness of HRV measure itself. We additionally tested the effects of the HRV window length on the reliability of functional connectivity and graph measures (SFigure 6). Our major conclusion, that reliability improved when sleepy volumes were excluded, was consistent across different window lengths. This improvement diminishes, however, if using a too short or too long window length. ICC of sleepy-0.5 condition decreased in general with longer window length, possibly due to the reduced volume number (Birn et al., 2013). Overall, the method proposed in this work is effective and efficient at improving test-retest reliability of rs-fMRI paradigm.

We additionally examined the effect of sleep on test-retest reliability during natural viewing paradigm. Unlike the effect on resting state measures, excluding volumes with higher HRV had very limited effect on the reliability of natural viewing data with as much as 50% volumes excluded regardless of the data length used (Figure 5B; SFigure 8). During movie viewing, cardiac autonomic activities are likely to be influenced by sustained attention and emotional saliency (Thayer and Lane, 2001) where high HRV does not necessarily reflect sleepiness. The ability of RMSSD to detect sleep is thus diminished. The test-retest reliability of connectivity measures is higher for natural viewing than resting state paradigm (Wang et al., 2017), which might be partially contributed by the high alertness during natural viewing.

There are several limitations to our study. Sleep is a complex physiological condition, and the use of single HRV measure for sleep detection might be oversimplified. In particular, HRV during movie viewing conditions is likely to be influenced by emotional responses rather than sleepiness. Therefore, the method we proposed here is a simple scheme to assess sleep and improve test-retest reliability for rs-fMRI paradigm, and our results on natural viewing should be considered with caution. Various methods have been previously used for sleep detection, such as subjective questionnaires, other physiological signals including EEG (Rechtschaffen and Kales, 1968; Iber et al., 2007), electrooculogram, electromyogram (Abbood et al., 2014), fMRI (Tagliazucchi et al., 2012; Tagliazucchi and Laufs, 2014), and more HRV measures (Sahayadhas et al., 2013). Advanced methods like machine learning have also been applied (Sahayadhas et al., 2012). With more advanced algorithm and/or additional physiological signals combined, it might be possible to further improve the accuracy of sleep detection, or expand such analysis to more complex conditions. In addition, it would be useful to examine whether HRV derived from pulse oximetry recording could provide similar results as ECG recording. Pulse oximetry is easier to implement and less affected by MR gradient artifact than ECG. While the smooth pulse waveform might offer less precision for peak detection, it has been shown to produce comparable HRV values as ECG (Iyriboz et al., 1991) and used to derive HRV values during rs-fMRI (Lv et al., 2015; Guo et al., 2016). Therefore, pulse oximetry might be used for sleep detection instead of ECG, which could be formally tested in the future studies.

## Author contributions

JW, conducted data analysis, wrote the manuscript; VN, collected the data, provided practical advice for data analysis; CG, initiatiated the study, designed the experiment, interpreated the results, edited the manuscript; LG and JH, provided administrative and material support.

## Funding

This work is supported by QIMR international fellowship, and NHMRC Project (Grant #1098407) and an NHMRC Career Development Fellowship (#1123674) to C.C.G., and the National Science Foundation of China (Grant #61522207) to JH.

### Conflict of interest statement

The authors declare that the research was conducted in the absence of any commercial or financial relationships that could be construed as a potential conflict of interest.
