# Supplementary material for: Improving the Test-Retest Reliability of Resting State fMRI by Removing the Impact of Sleep
Source: Front Neurosci. 2017 May 8;11:249. doi: 10.3389/fnins.2017.00249 (PMC5420587; doi:10.3389/fnins.2017.00249)
Supplement: Supplementary file 1 [file Table1.DOCX]

***Supplementary Material***

**Improving the test-retest reliability of resting state fMRI by removing the impact of sleep**

**Jiahui Wang, Junwei Han^*^, Vinh Thai Nguyen, Lei Guo, Christine Cong Guo^*^**

***** **Correspondence:** Christine Guo: [christine.cong@gmail.com](mailto:christine.cong@gmail.com), Junwei Han: [junweihan2010@gmail.com](mailto:junweihan2010@gmail.com).

# Supplementary Table

# Stable 1 One-tailed permutation tests of the difference in resting state reliability between the sleepy-0.5 or alert-0.5 and the random-0.5 conditions, based on CVI. Graph theoretical metrics were derived with Tr = 0.1. ICC and p values are listed for each condition. ICCs of random condition are indicated using upper and lower bounds marking 95 and 5 percentails of the random distribution, respectively. Nonsignificant results are in italic.

|  | | **Unit-wise** | | | **Scan-wise** | | |
| --- | --- | --- | --- | --- | --- | --- | --- |
|  |  | Random | Sleepy | Alert | Random | Sleepy | Alert |
| **Functional connectivity** | ICC | [0.321, 0.397] | 0.284 | *0.397* | [0.349, 0.624] | 0.343 | 0.653 |
|  | p | - | 0.001 | *0.0504* | - | 0.0432 | 0.0208 |
| **Clustering coefficient** | ICC | [0.314, 0.550] | 0.176 | 0.594 | [0.341, 0.628] | 0.318 | 0.678 |
|  | p | - | 0.0044 | 0.0082 | - | 0.031 | 0.0106 |
| **Degree centrality** | ICC | [0.338, 0.519] | 0.227 | 0.561 | [0.351, 0.625] | 0.334 | 0.663 |
|  | p | - | 0.0044 | 0.006 | - | 0.0362 | 0.0154 |
