# Supplementary material for: Improving the Test-Retest Reliability of Resting State fMRI by Removing the Impact of Sleep
Source: Front Neurosci. 2017 May 8;11:249. doi: 10.3389/fnins.2017.00249 (PMC5420587; doi:10.3389/fnins.2017.00249)
Supplement: Supplementary file 2 [file Image1.PDF]

*Supplementary Material*

# Improving the test-retest reliability of resting state fMRI by removing the impact of sleep

**Jiahui Wang, Junwei Han*, Vinh Thai Nguyen, Lei Guo, Christine Cong Guo***

*** Correspondence:** Christine Guo: christine.cong@gmail.com, Junwei Han: junweihan2010@gmail.com.

## Supplementary Figures

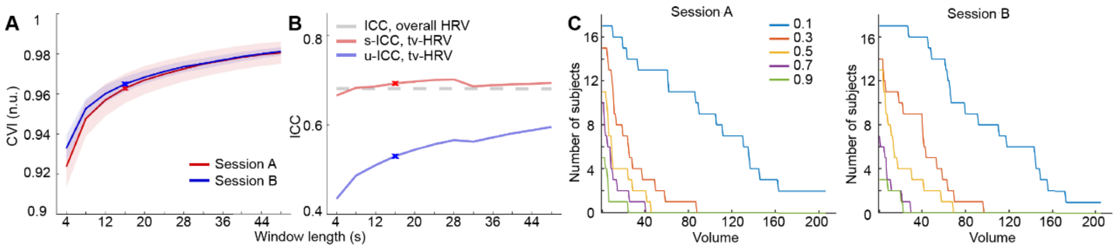


**SFigure 1** Heart rate variability analysis based on CVI. (**A**) Normalized CVI (CVI (n.u.), n.u. stands for 'normalized units') averaged across windows and subjects using different window length in both sessions. (**B**) ICCs of time-varying HRV (tv-HRV) using different window length at both unit- and scan-wise levels. The ICC of overall HRV is indicated by the dashed line. The window chosen to derive the main results (16s) is signified by crosses. (**C**) The number of subjects who successively stayed alert with scanning progression using a serial of selection threshold of sleepiness (signified by different colors). The appearance of consecutive 5 sleepy volumes was used as dropout criterion.

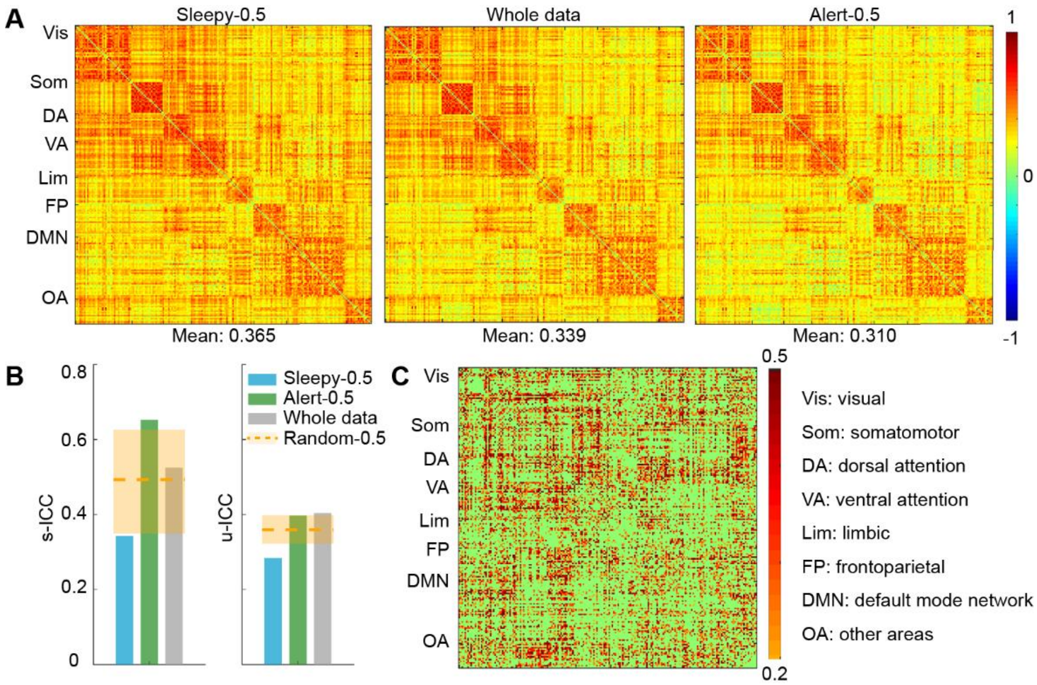


**SFigure 2** ROI connectivity matrix analysis of alert and sleepy conditions based on CVI. (**A**) Group-level connectivity matrices derived from the sleepy-0.5, whole-scan and alert-0.5 conditions during session A. ROIs were organized according to the 7-network system (Yeo et al.), as labeled on the left of each panel. The mean connectivity strength of each condition is indicated on the bottom of each matrix. The connectivity matrices in session B are very similar to those in session A, and thus not presented. (**B**) Functional connectivity ICCs during resting state at both scan- (left panel) and unit-wise (right panel) levels. Unit-wise ICC was averaged across ROI pairs. Orange dashed lines indicate the average ICC values of the random-0.5 conditions, and the shaded boxes indicate their distributions – upper and lower bounds marking the 95 and 5 percentiles, respectively. Values outside the boxes are significantly different from the random conditions (one-tailed permutation test, $p < 0.05$). (**C**) Unit-wise ICC differences between alert-0.5 and sleepy-0.5 conditions (warm color: alert-0.5 > sleepy-0.5; cool color: alert-0.5 < sleepy-0.5). Differences greater than 0.2 are displayed.

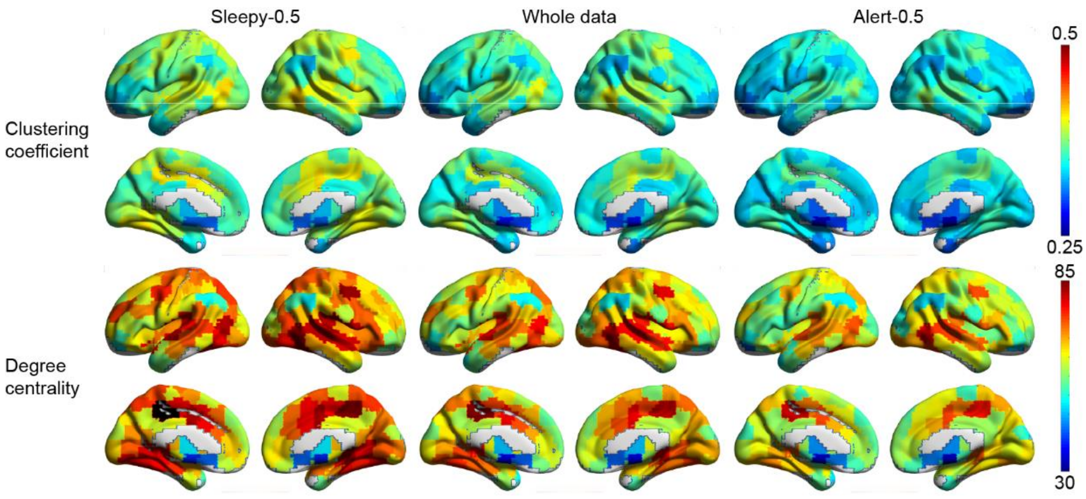


**SFigure 3** Graphic theoretical measure maps for session A, based on RMSSD. All results were derived at $T_r = 0.1$.

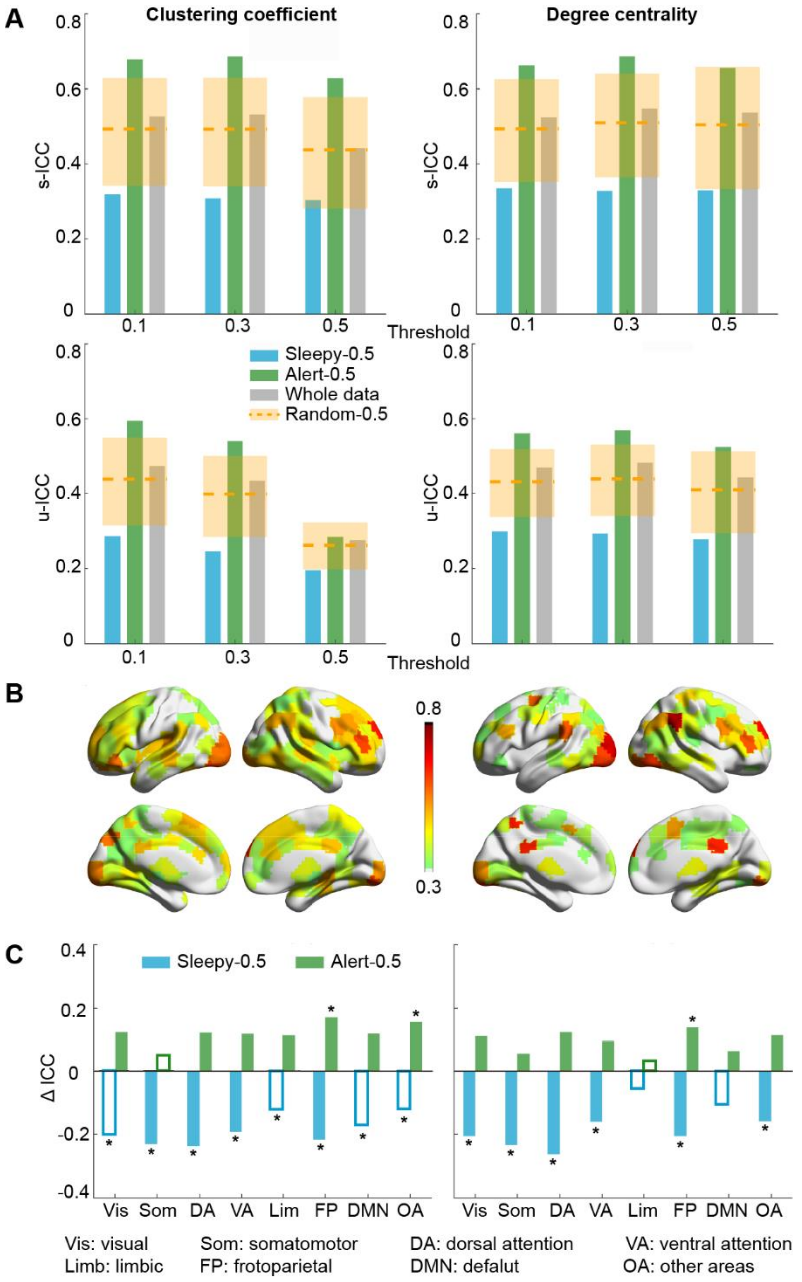


**SFigure 4** Test-retest reliability analysis using graph theoretical measures, based on CVI. (**A**) Average unit-wise (upper panel) and scan-wise (lower panel) ICCs during resting state across three thresholds ($T_r$ = 0.1, 0.3, 0.5). Orange dashed lines indicate the average ICC values of the random-0.5 conditions, and the shaded boxes indicate their distributions – upper and lower bounds marking the 95 and 5 percentiles, respectively. Values outside the boxes are significantly different from the random conditions (one-tailed permutation test, $p < 0.05$). (**B**) Unit-wise ICC differences between sleepy-0.5 and alert-0.5 conditions (warm color: alert-0.5 > sleepy-0.5; cool color: alert-0.5 < sleepy-0.5). Differences greater than 0.3 are displayed. (**C**) Unit-wise ICC difference between sleepy-0.5 or alert-0.5 and the whole data at network level, which is represented using mean across ROIs within each network. Solid bars indicate significant differences compared to the random-0.5 condition (one-tailed permutation test, FDR-corrected $p < 0.05$). Asterisks indicate ICC

changes over 30% relative to the whole data condition. Results in (**B**) and (**C**) were generated using $T_r = 0.1$.

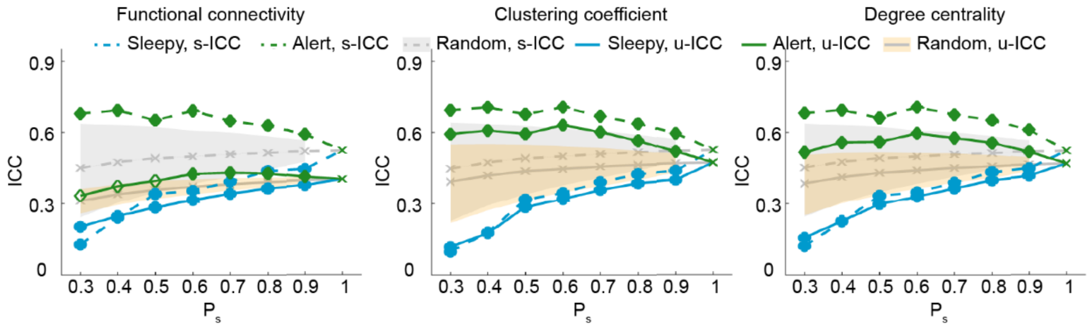


**SFigure 5** Test-retest reliability analysis using a serial of volume selection percentiles ($P_s$), based on CVI. The shades indicate the distribution of the ICCs derived from random condition – upper and lower bounds marking the 95 and 5 percentiles, respectively. Values outside the shades are significantly different from the random conditions, and represented using solid markers (one-tailed permutation test, $p < 0.05$). Results of clustering coefficient and degree centrality were obtained from $T_r = 0.1$.

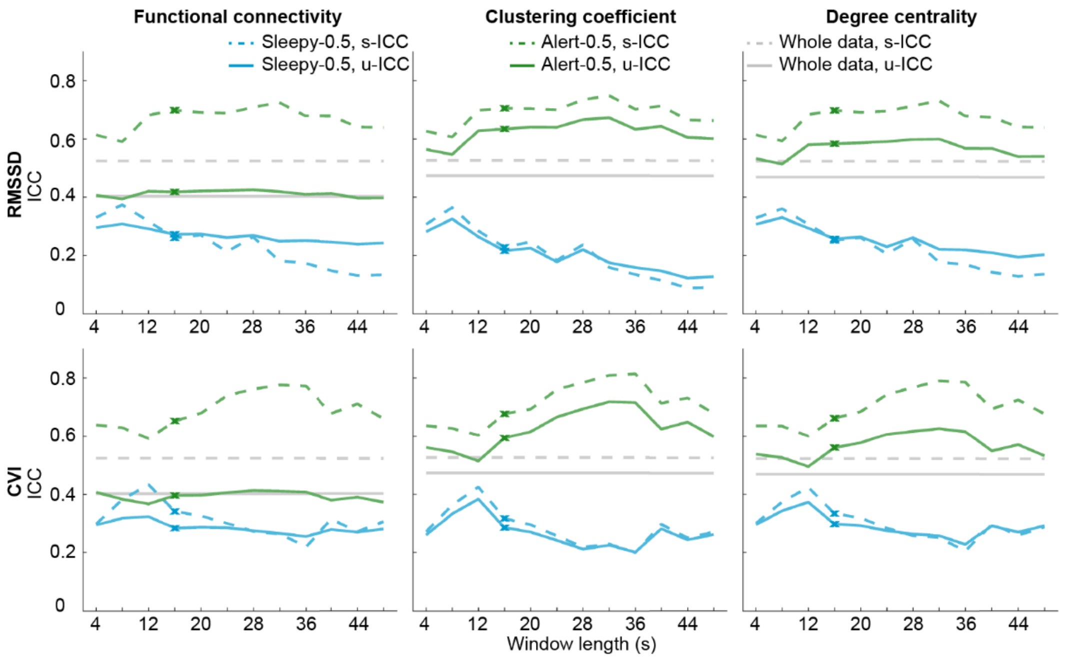


**SFigure 6** Test-retest reliability analysis using a serial of window lengths to derive time-varying HRV (RMSSD and CVI), for sleepy-0.5 (blue), alert-0.5 (green) and the whole data conditions (gray), respectively. Results of clustering coefficient and degree centrality were obtained from $T_r = 0.1$.

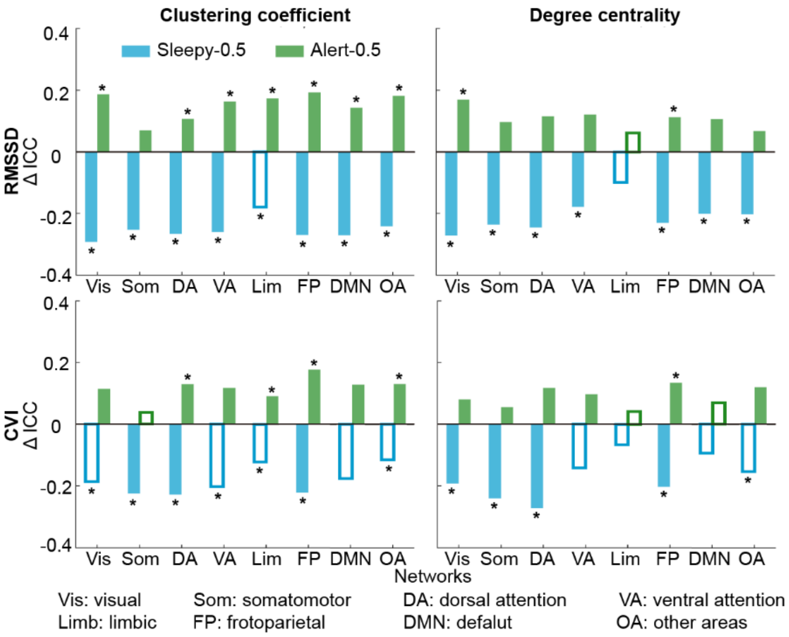


**SFigure 7** Unit-wise ICC difference between sleepy-0.5 or alert-0.5 and the whole data conditions at network level, which is represented using median ICC across ROIs within each network. Solid bars indicate significant differences compared to the random-0.5 condition (one-tailed permutation test, FDR-corrected $p < 0.05$). Asterisks indicate ICC changes over 30% relative to the whole data condition. The results presented were generated using $T_r = 0.1$.

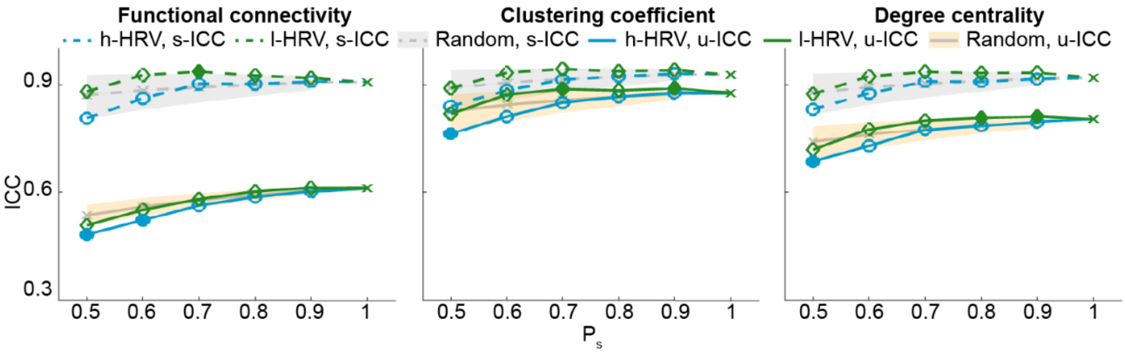


**SFigure 8** Test-retest reliability analysis of an 8-min segment of movie viewing data using a serial of volume selection percentiles ($P_s$), based on RMSSD. The shades indicate the distribution of the ICCs derived from random condition – upper and lower bounds marking the 95 and 5 percentiles, respectively. Values outside the shades are significantly different from the random conditions, and represented using solid markers (one-tailed permutation test, $p < 0.05$). Results of clustering coefficient and degree centrality were obtained from $T_r = 0.1$.
